# What Noseleaves Do for FM Bats Depends on Their Degree of Sensorial Specialization

**DOI:** 10.1371/journal.pone.0011893

**Published:** 2010-08-16

**Authors:** Dieter Vanderelst, Fons De Mey, Herbert Peremans, Inga Geipel, Elisabeth Kalko, Uwe Firzlaff

**Affiliations:** 1 Active Perception Lab, University of Antwerp, Antwerp, Belgium; 2 Institute of Experimental Ecology, University of Ulm, Ulm, Germany; 3 Smithsonian Tropical Research Institute, Balboa, Panama; 4 Lehrstühl für Zoologie, Technischen Universität München, Freising-Weihenstephan, Germany; University of Arizona, United States of America

## Abstract

**Background:**

Many bats vocalizing through their nose carry a prominent noseleaf that is involved in shaping the emission beam of these animals. To our knowledge, the exact role of these appendages has not been thoroughly investigated as for no single species both the hearing and the emission spatial sensitivities have been obtained. In this paper, we set out to evaluate the complete spatial sensitivity of two species of New World leaf-nosed bats: *Micronycteris microtis* and *Phyllostomus discolor*. From an ecological point of view, these species are interesting as they belong to the same family (Phyllostomidae) and their noseleaves are morphologically similar. They differ vastly in the niche they occupy. Comparing these species allows us to relate differences in function of the noseleaf to the ecological background of bat species.

**Methodology/Principal Findings:**

We simulate the spatial sensitivity of both the hearing and the emission subsystems of two species, *M. microtis* and *P. discolor*. This technique allows us to evaluate the respective roles played by the noseleaf in the echolocation system of these species. We find that the noseleaf of *M. microtis* focuses the radiated energy better and yields better control over the emission beam.

**Conclusions:**

From the evidence presented we conclude that the noseleaves serve quantitatively different functions for different bats. The main function of the noseleaf is to serve as an energy focusing mechanism that increases the difference between the reflected energy from objects in the focal area and objects in the periphery. However, despite the gross morphological similarities between the noseleaves of the two Phyllostomid species they focus the energy to a different extent, a capability that can be linked to the different ecological niches occupied by the two species.

## Introduction

Bats, with the exception of flying foxes (Pteropodidae), use a sophisticated biosonar system to navigate and forage in dark, and often complex, environments [1], [2]. For obvious reasons, spatial hearing is of capital importance for these animals. It is known that the outer ears of bats, by means of spatial filtering, generate cues allowing localization of sound sources (origins of echoes) in azimuth and elevation [3]–[6]. This has been confirmed in theoretical analyses [7], [8]. The most direct way of studying the spatial cues that are generated by the outer ears is by considering the head related transfer function (HRTF, [9]). The HRTF fully describes the spatial filtering introduced by a bat's ears and head [3], [10]–[12]. To evaluate the role played by particular subcomponents of the pinnae HRTFs have also been obtained after deformation of the external ears, for example, after removal of the tragus, e.g. [4], [10], [11].

Sonar being an active sense, the HRTF is only part of the story (see [5], [13] and references herein) as the spatial sensitivity of the complete sonar system is also determined by the radiation pattern of the emission subsystem. Measuring the emission radiation pattern of bats has turned out to be more difficult than assessing the HRTF. The latter can be measured by fitting a microphone into the ear canal of a freshly sacrificed bat (see [11] for an overview of the technique). In contrast, the animal needs to be alive for recordings of the emission radiation pattern and needs to be stimulated in order to emit calls. Electrical stimulation of the bat's brain is the standard technique to elicit calls from restrained and usually sedated bats [14], [15]. The difficulty of this procedure is at least partially responsible for the very small number of detailed radiation patterns reported in the literature. Recording the emission radiation pattern of awake bats requires the usage of an array of microphones in combination with a head-orientation sensor and, ideally, naturally behaving animals. As this type of equipment is only now becoming available (e.g. [16]), this explains why only very few data on radiation patterns are available (see [17] for early references). In order to overcome the methodological difficulties associated with measuring both the radiation pattern and the HRTF of bat species, we use recently developed simulation methods [18] in combination with morphological data of complete bat heads obtained by microCT [19]. Several authors have proposed computational methods to simulate the HRTF instead of measuring it because of the increased flexibility [19]–[23]. For example, models can be easily deformed to evaluate the contribution of certain morphological features to the HRTF without hurting or disturbing live bats [19], [23]. Simulations also permit to describe the spatial sensitivity patterns with a higher resolution than has been practical using traditional techniques.

Many bats that emit calls through their nose feature a conspicuous noseleaf. It has been argued that in FM bats (Phyllostomidae) the emission radiation pattern introduces considerable spatial variability in the echo spectrum that may provide additional spatial localization cues in combination with the HRTF [24]. In the absence of a measured HRTF for *Carollia perspicillata*, the bat species that has been used in the latter study, this conclusion was based solely on the analysis of the emission radiation pattern. However, as acknowledged in that study, spatial cues can only arise from the combination of the spatial characteristics of both the hearing and the emission subsystem. Since it is not clear which particular cues introduced by the emission subsystem are still salient when analyzing the spatial sensitivity of the complete sonar system, we argue that in the case of *C. perspicillata* the specific contribution of the emission radiation pattern to the overall spatial sensitivity remains as yet unknown. A similar concern can be raised with respect to the simulation studies of how the noseleaf affects the emission radiation pattern of *Rhinolophus rouxi* a member of the horseshoe bats (Rhinolophidae) that emits constant-frequency (CF) calls [22], [25]. While the HRTF of this bat had been measured before by [3], in these studies again no results are included on the combined spatial sensitivities of the emission and the hearing subsystems making it difficult to ascertain what impact the described mechanism will have on the overall spatial sensitivity of that sonar system. In addition, both noseleaf carrying groups, phyllostomids and rhinolophids, do not only differ in the structure of their noseleaves but also in the design of their echolocation calls (FM versus CF calls).

**Figure 1:**
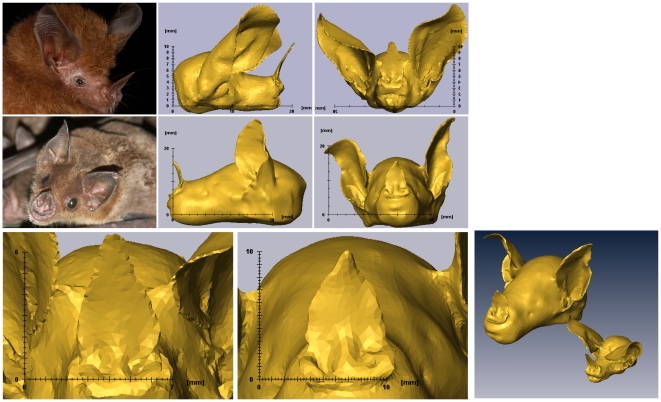
3d Models of the bats. Images of the bat species and the 3D mesh models used in the acoustic simulations. Top row: *M. microtis*, Middle row: *P. discolor*. Bottom row: Details of noseleaves and a figure containing both models to the scale for comparison of size.

We argue that the morphology acquisition and simulation technique proposed in this paper addresses these remaining questions by allowing efficient and detailed characterization and comparison of both the emission radiation patterns and the HRTF's of noseleaf carrying bats. We demonstrate this approach by applying it to two species of FM bats, *Phyllostomus discolor* and *Micronycteris microtis* (see Figure 1 for photos). In order to evaluate the function of the most prominent part of the noseleaf i.e., the lancet, we compare the spatial sensitivity of the original models with versions of the models from which the lancet was removed (see Figure 1). In addition, Phyllostomidae have a flexible lancet that can be moved back and forth, possibly allowing active steering of the emission beam [24], [26], [27]. Hence, we also quantify the role of a deformable lancet in the spatial sensitivity of the complete sonar systems of these animals by bending it and evaluating the resulting changes.

We selected *M. microtis* and *P. discolor* for this study as we suspected that the functional role of the lancet in FM bats might be correlated with their degree of sensorial specialization as has been suggested for call structure [28], [29]. From an ecological point of view, *P. discolor* and *M. microtis* are particularly interesting species because they belong to the same family (Phyllostomidae). They are similar in general morphology but differ considerably in their foraging behaviour and diet. *M. microtis* (body length: 35–51 mm, weight: 4–9 grams, [30]) is a bat that specializes in gleaning from flight, thereby taking rather large and non-flying insects from vegetation within very small hunting grounds in the forest [30], [31]. In contrast, *P. discolor* (body length: 89–109 mm, weight: 40–44 grams, [32]) is an omnivorous bat roams widely and feeds on a wide range of food items including nectar, pollen, flowers, fruits and occasionally a few insects depending on locality and season [32], [33]. Although both species use echolocation for orientation and navigation, *P. discolor* uses additional cues, mainly olfaction, for detection, classification and, in part, localization of food. Furthermore, this bat has been found to hunt making use of visual cues [27], [32]. In contrast, *M. microtis* can perform all of those tasks by echolocation alone [30].

Based on a detailed evaluation of the spatial sensitivity of the complete sonar systems (i.e. the combination of the hearing and emission spatial sensitivity) we conclude that the noseleaves play different roles in these animals' perceptual systems. In addition, we interpret the differences in the roles played by the noseleaves in the context of the different ecologies of these two species. Indeed, the exact role of the noseleaf seems to depend to a large degree on the specific natural history of the species under study. Our data show that studying the role of the noseleaf in related species with rather similar morphologies but that inhabit different ecological niches allows to discover fine-grained differences that have been overlooked so far.

## Results

### Spatial sensitivity patterns

**Figure 2:**
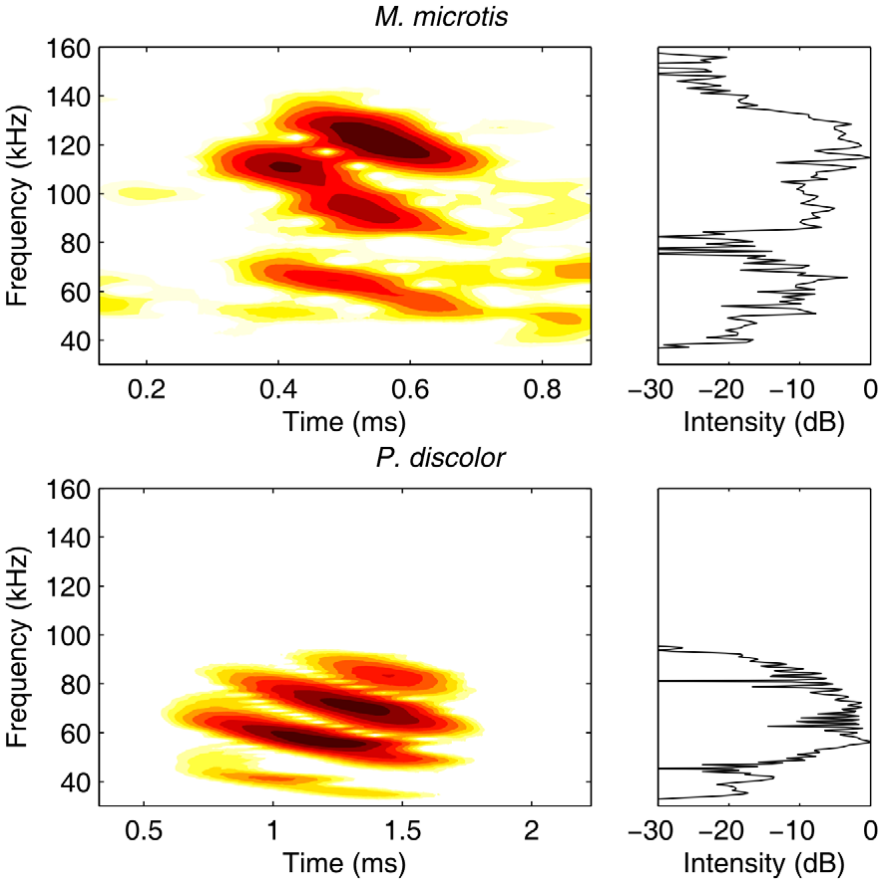
Spectrograms of the calls of *M. microtis* and *P. discolor*. Top: Spectrogram and spectrum of a search call of *M. microtis*. Bottom: Spectrogram and spectrum of a search call of *P. discolor*.

Based on the spectrum of the calls of *M. microtis* recorded from wild-caught animals (Figure 2 and [30]), we evaluated the spatial sensitivity of this sonar system in the range from 50 to 150 kHz. *P. discolor* emits most energy in a frequency band between 30 and 95 kHz (Figure 2 and [32]). Therefore, we limit our analysis to this range. We report on the left ear of *P. discolor* and the right ear of *M. microtis* (see methods for details). However, we mirrored the data of the right ear of *M. microtis* to make the data congruent with that of *P. discolor*.

**Figure 3:**
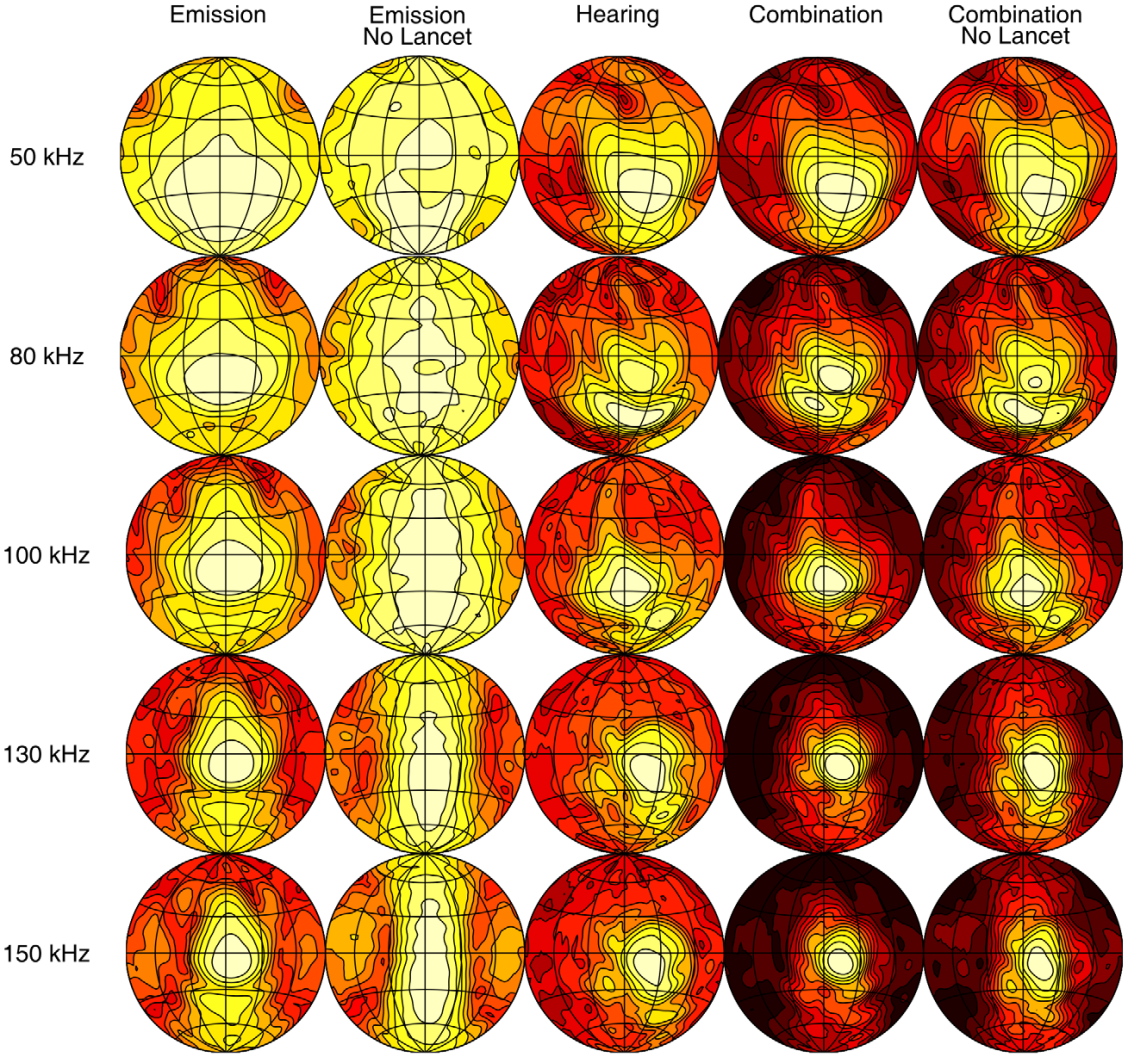
Spatial sensitivity patterns of *M. microtis* at selected frequencies. The different columns give the spatial sensitivity for different versions of the (sub)model(s). The contour lines are spaced 3 dB apart. All plots are normalized such that the maximum is 0 dB.

**Figure 4:**
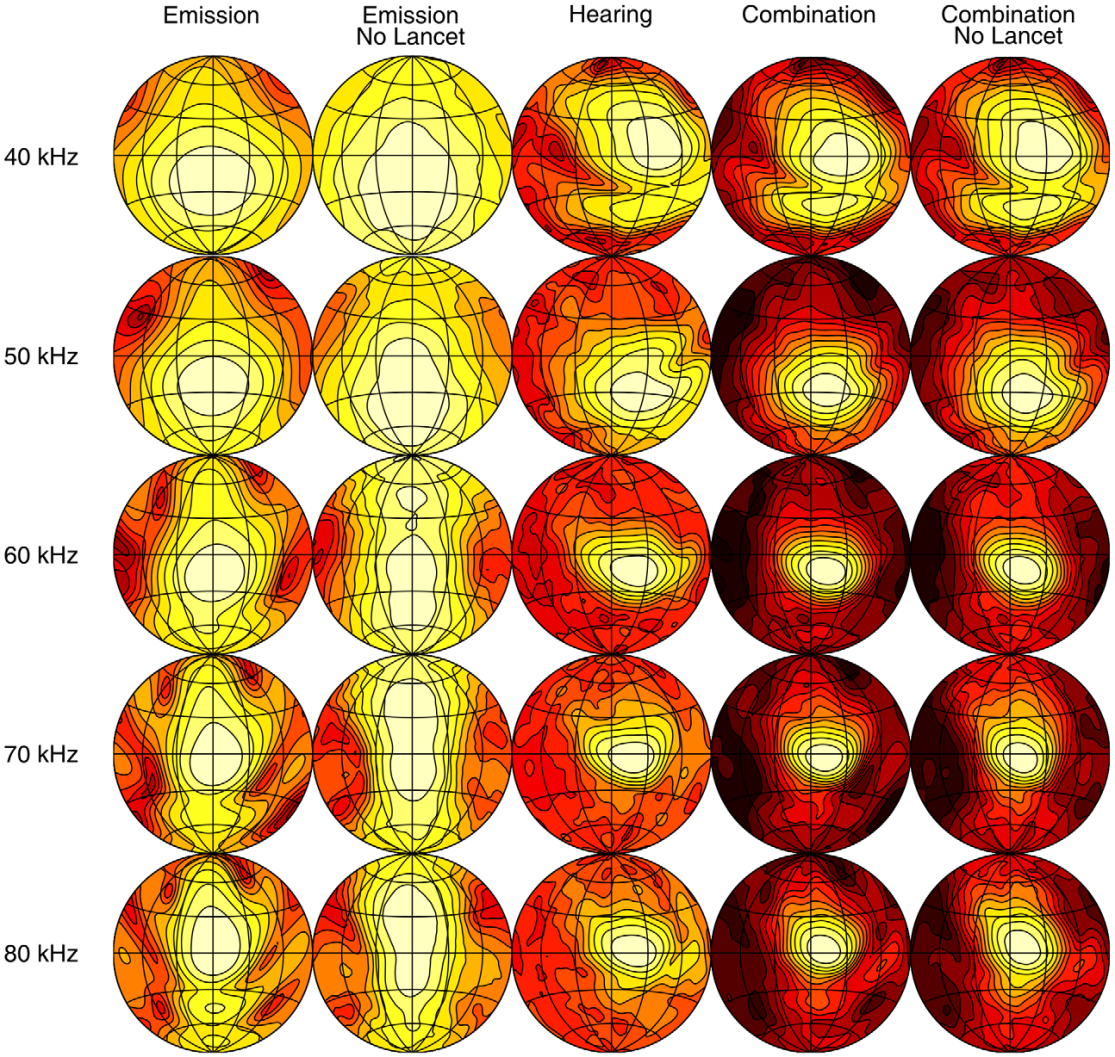
Spatial sensitivity patterns of the *P. discolor* at selected frequencies. The different columns give the spatial sensitivity for different versions of the (sub)model(s). The contour lines are spaced 3 dB apart. All plots are normalized such that the maximum is 0 dB.

The simulated spatial sensitivity patterns are displayed as a set of shaded contour plots (Figures 3 and 4). These show the relative intensity across the frontal hemisphere for a representative set of frequencies contained in the emission spectrum of the two bat species for the emission with and without lancet, the HRTF and the complete sonar system with and without the lancet. The shading corresponds to relative intensity, dark regions being areas of low intensity and light regions of highest intensity. The first contour encircles the region of maximum intensity and the remaining contour lines are plotted at 3 dB decrements in intensity.

### Agreement between simulations and measurements of noseleaf carrying FM bats

We note that the simulated spatial sensitivity patterns are in good qualitative agreement with those available from previous studies based on standard measurement techniques. A detailed comparison of the simulated and measured HRTF of *P. discolor* can be found in [19]. As measured emission patterns are unavailable for neither of the two FM bat species we compare the qualitative features of the simulated emission radiation patterns with the *C. perspicillata* measurements presented in [24].

As expected, the emission patterns are frequency dependent with a distinct broadening in both horizontal and vertical dimensions at lower frequencies. The main beam of the emission patterns is directed forward pointing below the horizon for the lower frequencies in the call spectrum and sweeping upward as frequency increases, with half-amplitude beamwidths ranging between 45 (80 kHz) and 25 (150 kHz) for *M. microtis* and between 45 (50 kHz) and 28 (80 kHz) for *P. discolor*. These numbers are in full accordance with those reported for *C. perspicillata* i.e., half-amplitude beamwidths ranging between 40 (70 kHz) and 28 (90 kHz). In addition to the main beam, both emission patterns also contain minima and sidelobes in the horizontal dimension at the high frequency end of the call spectrum as well as a downward aimed secondary lobe below the main beam of similar prominence as the ventral lobe found in the *C. perspicillata* measurements. Hence, there is good qualitative agreement between the simulated emission patterns of the intact noseleaves of *M. microtis* (Figure 3), *P. discolor* (Figure 4) and *C. perspicillata*.

In [24] the emission pattern of an intact noseleaf is compared with that of a noseleaf with the tip of the lancet cemented back to the head. From this comparison it is concluded that the lancet in *C. perspicillata* acts by directing sound in the vertical dimension but plays little or no role in directing sound in the horizontal dimension. A pronounced broadening of the emission pattern in the vertical dimension in combination with nearly unchanged horizontal dimensions is also observed in the simulation results for both *M. microtis* and *P. discolor* when we remove the lancet from the model by virtually cutting it off at its base. Hence, this simulation study further confirms the hypothesis that the lancet affects mostly the vertical dimension of the emission pattern features while leaving the horizontal dimensions of the emission pattern features unchanged.

**Figure 5:**
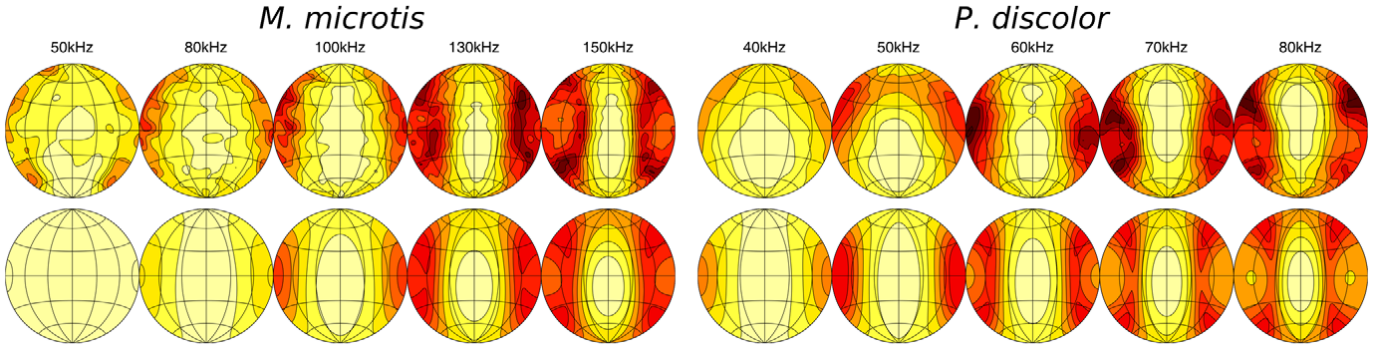
Comparison of the emission patterns without lancet with that of two baffled pistons. Top rows: The emission patterns of *M. microtis* (left) and *P. discolor* (right) without lancet. Bottom rows: The emission patterns of two baffled pistons with properties fitted to the emission patterns of the models without lancets.

A further confirmation of this conclusion is present in the good qualitative correspondence of the emission patterns in the ‘removed lancet’ condition and a simplified theoretical model [34] consisting of two baffled pistons positioned at the nostrils as shown in Figure 5.

The properties of the two baffled pistons are fitted to the emission patterns of the models without lancets. The emission pattern of two baffled pistons is derived analytically in [34]. The three free parameters were the rotation in azimuth and elevation of the pistons and their radius. The fitting procedure consisted of an extensive search for the best fit in a wide range of reasonable parameter settings.

### Target localization cues

From the results shown in Figures 3 and 4 it is apparent that despite the obvious differences between the emission patterns for the noseleaf with and without lancet the spatial sensitivity patterns of the complete sonar systems look very similar. However, as no generally agreed upon distance measure for spatial sensitivity patterns has been proposed so far the significance of the observed differences in the complete spatial sensitivity patterns with and without lancet is difficult to assess. Hence, we propose to evaluate whether the observed differences between the spatial sensitivity patterns have functional significance in terms of their impact on expected target localization performance. To this end, we compare the impact on target localization of the observed differences due to removal of the lancet with that introduced by removal of the tragus. We chose this particular manipulation of the spatial sensitivity pattern as a reference since it is well documented that removing or bending the tragus changes the HRTF of bats [11] and interferes with the echolocation performance of both FM bats in experimental setups [35] and naturally behaving bats [6].

**Figure 6:**
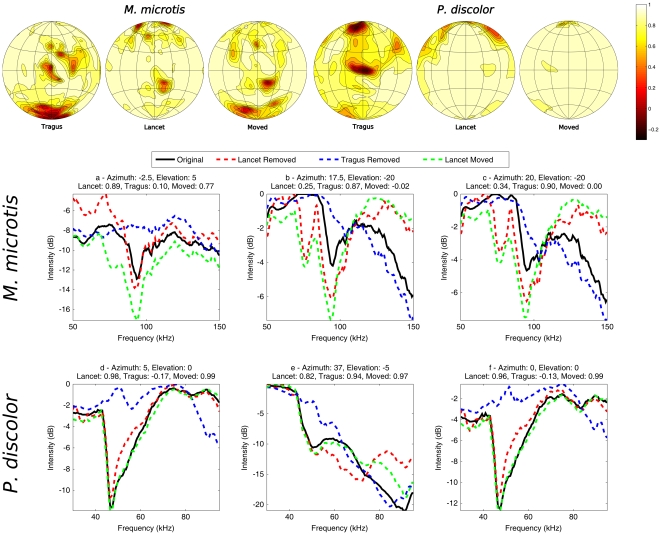
The effect of the removal of the tragus and lancet on the spetial sensitivity. Top figures: The correlation between the spectra of the complete sonar system with, without lancet or tragus and a bent lancet as a function of azimuth and elevation. Left column: *M. microtis*, Right column: *P. discolor*. Subfigures a–d give examples of the spectra of the complete sonar system for *M. microtis* and *P. discolor* on which the correlations in the top row are based. The azimuth and elevation location of the example spectra are indicated above each subfigure. Separate traces are drawn for the models without lancet (red), without tragus (blue) and with a bent lancet (green). The correlations between the spectra without lancet or tragus and the original spectrum are indicated in the title of each subfigure.

We calculate for each azimuth-elevation position in the frontal hemisphere the Pearson correlation between the echo spectra for the models with and without lancet (for sample spectra see Figure 6a–f, note that Figure 6 also contains data generated with models with a moved lancet. This data is discussed later). The spectra we use are the combination of the emission patterns and the HRTF. We compare these correlations with the correlations between the echo spectra for the models with and without tragus.

The results of this analysis are depicted in the top row of Figure 6. As the correlation measure is a similarity measure the areas of high correlation correspond with regions where the echo spectra are invariant to removal of the lancet or tragus whereas low correlation values point to regions where the echo spectra are particularly sensitive to these manipulations of the sonar apparatus morphology. This figure reveals that removal of the tragi in both species influences the complete spatial sensitivity pattern most in an area around zero elevation and zero azimuth. For *M. microtis* there is a secondary area of high susceptibility around 15 degrees azimuth and 15 degrees elevation. In the case of *P. discolor* the central region of low correlation corresponds well with the changes to the HRTF introduced by removal of the tragus as reported by [11].

In *P. discolor* the differences between the original echo spectra and those generated without lancet are comparable to those introduced by tragus removal in the periphery only. However, for *M. microtis* apart from the periphery there is also an area around −20 elevation where the spectral changes due to lancet removal and tragus removal are of comparable magnitude. Interestingly, in *M. microtis*, the area where the tragus and the noseleaf influence the complete spatial sensitivity pattern seem to be complementary.

To further illustrate the changes in the complete spatial sensitivity introduced by the tragus and the lancet of the two species, spectra corresponding to selected locations are drawn in Figures 6a–f. These locations are chosen as the original and the altered spectra do not correlated well at these locations indicating a change in the spectra due to altering the model(s). Figures 6a & d reveal that removing the tragus causes, for central positions, a spectral notch disappears for both species. However, for the same positions, removing the lancet has little influence on the spectra. In contrast, removing the lancet in *M. microtis* introduces a new spectral notch and enhances an existing one in the area around 20 azimuth and −20 elevation (Figure 6c). For *P. discolor* even in the small region in the central area where the correlation between the spatial sensitivity of the model with and without lancet is the lowest, the spectra are not much altered by removal of the lancet (Figure 6d).

### Spatial distribution of energy and clutter rejection

**Figure 7:**
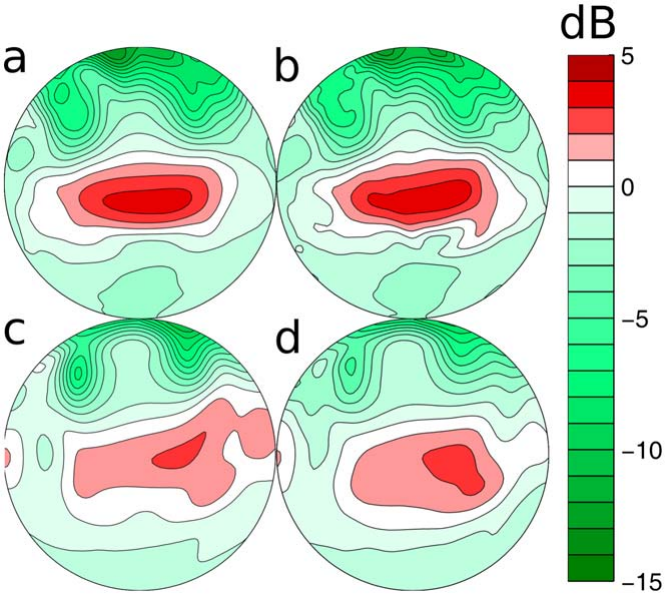
The effect of the removal of the tragus and lancet on the energy distribution. The ratio between the energy emitted in each direction with the lancet present versus with the lancet removed in the two species under study. a–b: *M. microtis*, c–d: *P. discolor*. The left column (a & c) depicts data based on emission patterns only. The right column (b & d) depicts data based on the complete spatial sensitivity patterns (combined emission pattern and HRTF). The contour lines are spaced 1 dB apart.

The results presented above indicate that removal of the lancet causes different changes in the spatial sensitivity patterns of the complete sonar systems of the two species. In particular, the changes for *P. discolor* are smaller than for *M. microtis*. In this section we evaluate what effect the lancet has on the spatial distribution over the frontal hemisphere of the emitted energy and thus the echo signal to noise ratio (SNR). Indeed, the removal of the lancet results, for both species, in an emission main lobe with increased vertical dimension resulting in the distribution of the emitted sound energy over a larger area (Figures 3 and 4). We calculate the ratio of the radiated energy i.e., the square of the spatial sensitivity pattern integrated across frequency, with and without lancet for both species for each azimuth and elevation position. We plot these results in dB as a function of location in Figure 7.

In *M. microtis* (Figure 7 top row) it is shown that due to the presence of the lancet the energy emitted in the frontal direction (around zero azimuth and elevation) is increased by about 4 dB. In the periphery, the lancet reduces the amount of allocated energy substantially (down to −14 dB). While the overall pattern of energy redistribution is similar for both species, in *P. discolor* (Figure 7 bottom row) the redistribution in the frontal direction (2 dB) at the expense of the periphery (−8 dB) is less pronounced. In both species, especially the high elevation positions (above 30) receive less energy due to the inclusion of the lancet.

The relocation of energy by the lancet of both species is not only clearly visible in the emission patterns but also in the complete spatial sensitivity patterns. The redistribution of the emitted energy by lancet in the emission patterns, changes the spatial sensitivity of the complete sonar system of both species. Indeed, both systems are rendered more sensitive to the central region and less to the periphery by the presence of the lancet.

Reducing the amount of energy that is radiated towards the periphery potentially reduces the strength of clutter echoes (Figure 7 righthand side). Indeed, the bat can better isolate the objects it wants to ensonify if its beam is less wide. Clutter echoes will reduce the ability of the bat to locate and recognize objects because overlapping echoes interfere with the echo of interest (i.e. backward masking [28]). This interference causes the spectrum of the target echo to be altered. To test the influence the lancets have on the reduction of clutter, we simulate their effect on the spectrum of an object at zero azimuth. The elevation position of the object is taken as the elevation for which the sonar system of each bat is most sensitive. For *M. microtis* this is −12.5. For *P. discolor* the chosen elevation is −7.5. We generate clutter echoes overlapping with the target echoes coming from outside the center region, i.e. we simulate peripheral clutter echoes. We vary the region we consider as the center region from 30 to 80. For example, when we consider 50 as the central region, we simulate clutter echoes coming from at most 50 from the azimuth and elevation position from which the target echo comes, i.e. clutter echoes from a cone with an opening of 100. Also, the strength of the clutter echoes is varied between −20 to 20 dB. We calculate the variability in the resulting echo spectrum (see Materials and Methods). This uncertainty about the spectra of the returning echo poses a fundamental limit on the performance of bats to locate and recognize targets [7].

**Figure 8:**
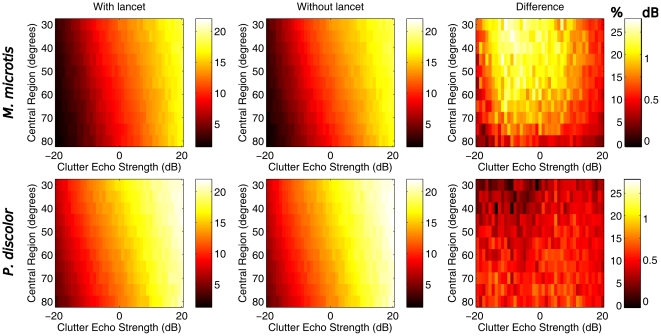
The reduction of clutter interference by the lancet. The simulated error variance (averaged across frequencies) in the spectra of 1000 target echoes with 1 to 4 overlapping clutter echoes. Top Row: *M. microtis*, Bottom row: *P. discolor*. Traces are drawn for the two species and the models with and without lancet. Left and middle columns: variance in the spectra for the model with and without lancet as a function of central region size and clutter echo strenght. Right column: difference in the error variance between the models. There are two scales on these plots. The scale is both expressed as the absolute reduction and as the percentage of the noise variation for the model without lancet is reduced by the lancet.

Figure 8, shows the variation (see Methods and Materials for details) on the spectra of the simulated target echo averaged across frequency. These plots show that, as the strength of the clutter echoes increases, the variability in the received spectrum increases also. We calculate the difference in variation between the sonar systems with and without lancet. In *M. microtis* the reduction in variation in the spectra due to the presence of the lancet is markedly larger than in *P. discolor*. Even without the lancet *M. microtis*, suffers less than *P. discolor* (without noseleaf) from the interference from clutter echoes. However, on top of this, the influence of clutter echoes is reduced more in *M. microtis* by the presence of the lancet than in *P. discolor*. Expressed in percentages, the lancet of *M. microtis* reduces the effect of clutter echoes by up to 25% in the presented simulations.

### Emission beam steering by lancet movement

The lancets of both *M. microtis* and *P. discolor* [27] have been observed to move. Lancet movements and their possible role in emission beam steering by *C. perspicillata* have also been briefly reported on in [24]. However, no quantitative data are available on the lancet movements in any of those species. To the authors' best knowledge lancet movements have been formally documented only in *Macrophyllum macrophyllum* [26]. However, the emission pattern of this species has not been measured yet. Hence, it has not been possible to study in detail the effect of lancet movements on the emitted sound field thus far.

**Figure 9:**
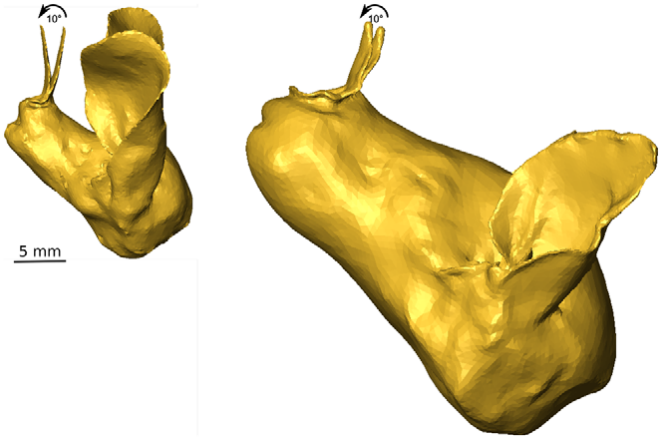
Details of the models illustrating the movement of the lancets. Left: *M. microtis*, Right: *P. discolor*. The arrows indicate the direction in which the noseleaves were bent. The scale bar represent 5 mm. The scale is the same for both models.

The finding that the removal of the lancets of *M. microtis* and *P. discolor* animals has a significant effect on their emission patterns leads to the hypothesis that movement of the lancets might be used by these bats to actively control their spatial sensitivity patterns in order to reallocate energy over space or adaptively introduce spectral localization cues in particular regions of interest. To test these hypotheses, we rotate the lancets of the mesh models of the bats (see Figure 9) and simulate the resulting emission patterns. The lancets both of *M. microtis* and *P. discolor* are rotated forward by about 10 degrees. As illustrated in Figure 9 the lancet is rotated rigidly around the axis constituting the intersection between the plane of the lancet and the base of the noseleaf surrounding the nostrils. In the absence of more specific information about the actual noseleaf deformations occurring in both bat species the proposed rotations of the lancets are a first approximation of the lancet movements that have been observed.

**Figure 10:**
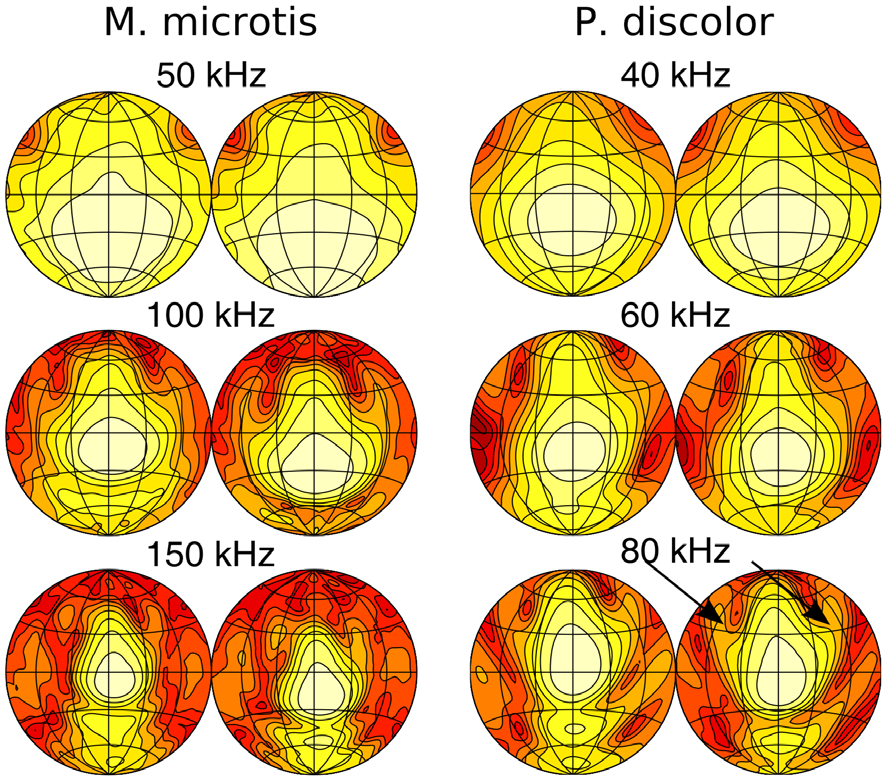
The emission patterns with the lancets bent forward of *M. microtis* and *P. discolor* for selected frequencies. The left column for each animals gives the emission patterns for the lancets in the original position. The arrows indicate the sidelobes generated by the movement of the noseleaf of *P. discolor*.

Figure 10 illustrates the emission pattern of both species with the lancet rotated. These figures indicate that rotating the noseleaf shifts the emission beam to a lower elevation position across frequencies. For the higher frequencies, diffraction around the noseleaf of *P. discolor* seems to be creating sidelobes (see arrows in Figure 10). Moving the lancet, not only affects the emission pattern, it also changes the spatial sensitivity of the complete sonar system. Hence, this change in the spatial sensitivity might lead to enhanced localization cues. To gauge the potential significance of the changes to the spatial sensitivity induced by the bent lancet we correlated the altered spatial sensitivity with the original spatial sensitivity (as done for the removal of the lancet). The results, depicted in Figure 6, show that bending the lancet, for certain regions of the frontal hemisphere, indeed results in spectral changes with similar effect on the correlation as produced by tragus removal (see Figures 6e–f for examples).

**Figure 11:**
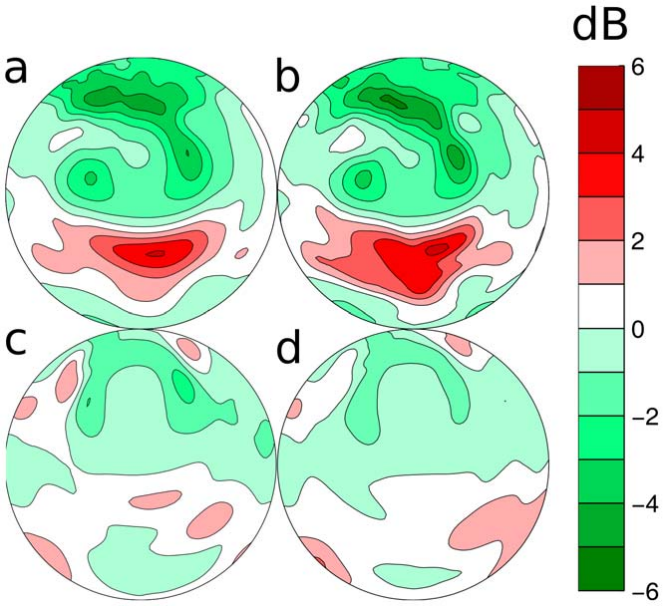
Energy redistribution by the movement of the lancet. This figure depicts the ratio between the energy radiated in each direction with the lancet in the original position and the lancet bent forward. a–b: *M. microtis*, c–d: *P. discolor*. The left column (a & c) depicts data based on emission patterns only. The right column (b & d) depicts data based on the complete spatial sensitivity patterns (combined emission pattern and HRTF). The contour lines are spaced 1 dB apart.

Figure 11 depicts the ratio between the energy emitted in each direction by the models with the rotated lancets and the models with the lancets in the original position. From these plots it is apparent that the proposed movement of the lancet also shifts the allocated energy to a lower elevation position in both *M. microtis* and *P. discolor*.

## Discussion

### Changes in spatial sensitivity

Based on measurements of the emission pattern of *C. perspicillata*, it has been argued that the emission radiation pattern, as shaped by the lancet, might introduce spectral cues that increase the spatial acuity with which bats can echolocate [24]. However, as acknowledged by the authors of that study, this hypothesis can not be tested without knowledge of the HRTF as only the cues present in the complete spatial sensitivity pattern are available to the bat's sonar system.

Given the lack of decisive studies on the interaction between the emission pattern and the spatial hearing sensitivity of FM bats carrying noseleaves, we evaluated the spatial sensitivity pattern of the complete sonar system with and without the lancet to improve our understanding of the role of the noseleaf in the generation of spectral cues for localization. For both species removing the lancet clearly affects the emission pattern. However, after combining the emission radiation pattern with the HRTF, the spectral effects of the removal of the lancet are greatly attenuated.

For most locations in the frontal hemisphere, the spatial sensitivity pattern of the whole sonar system does not seem substantially altered by the removal of the lancet in the species under study. To address methodological questions about what changes in the spatial sensitivity pattern might constitute a significant change to the bats we have compared the spectral changes due to the removal of the lancet with those due to the removal of the tragus. The results indicate that lancet removal and the associated changes in the complete spatial sensitivity pattern are likely to have only limited functional influence in *P. discolor*. Only in the periphery are the dissimilarities between the original echo spectra and those generated without lancet as large as they get after tragus removal. Interestingly, for *M. microtis* there is an area around −20 elevation where the dissimilarity is sufficiently high to suggest a functional effect. On the one hand, these changes in spatial sensitivity might either introduce new spatial localization cues or augment the robustness of existing cues against noise or unknown reflector filtering. On the other hand it is also conceivable that the changes to the spatial sensitivity due to the presence of the lancet observed in *M. microtis* are behaviorally irrelevant. The same might hold for the spectral effects due to bending the lancet. In the absence of an agreed upon model to evaluate the functional role of these observed changes we propose that behavioral experiments comparing the localization performance of bats with an intact noseleaf and a deformed one e.g., lancet bent backwards, should be performed to verify whether the changes in the spatial sensitivity due to the lancet in *M. microtis* are behaviorally relevant.

Furthermore, given the results on the *P. discolor* our results indicate that the conclusions on the functionality of the lancet in *C. perspicillata* [24] can not be considered conclusive in the absence of an analysis of the combination of the HRTF and the emission pattern.

### Spatial distribution of energy

While the noseleaf has only limited impact on the spatial sensitivity pattern at each single frequency both bats in our study emit broadband multi-harmonic FM signals. Integrating the differences in spatial sensitivity pattern across frequencies reveals that the noseleaf systematically redistributes the energy in space. Hence, the noseleaf helps in enhancing the echo signal to noise ratio attained by the bats in a confined region that is of most interest to them i.e., a focal area.

*M. microtis* uses higher frequencies than *P. discolor*. This is most likely an adaptation to hunting in cluttered environments. Indeed, even bats without noseleaves using higher frequencies are better able to cope with backward masking (*Myotis* spp., [29]). On top of its usage of higher frequencies, *M. microtis* carries a noseleaf that is about the same length as the noseleaf of *P. discolor*. Therefore, the noseleaf of *M. microtis* is longer with respect to the wavelengths in its call than the noseleaf of *P. discolor* (see Table 1). The effect of this is that, in particular in the vertical dimension, the energy redistribution is more pronounced in *M. microtis* than in *P. discolor* (Figure 7).

**Table 1:** Size of the lancets compared to the wavelengths.

	*M. microtis*		*P. discolor*	
	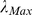	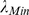	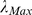	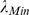
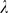 (mm)	6.86	2.29	11.43	3.61
Ratio Width	0.55	1.66	0.54	1.73
Ratio Height	1.00	3.01	0.64	2.03

The ratios between the sizes of the noseleaves (width and height) of both species and the wavelengths of the frequencies in their calls.

We conjecture that providing a spatially dependent energy gain is the most important function of the noseleaf as it results in a number of benefits. First, by focusing the energy into a small focal area, the bat will be able to detect weaker echoes from targets in this focal area than it would otherwise. Secondly, the noseleaf enhances the difference in energy between echoes coming from a frontal region of interest and those coming from elsewhere. Hence, in combination with the reduced sensitivity of the ears for peripheral directions, the noseleaf acts to reduce the number of detectable (clutter) echoes received from directions that are of less interest to the bat thereby implementing an early spatial selection mechanism [36]. We have confirmed this by simulating clutter echoes and evaluating their effect on the spectrum of an echo coming from a target at zero azimuth and zero elevation. Our results show that the lancets are most effective in reducing the influence of clutter echoes originating from about 40 from the center of the frontal hemisphere. The lancet of *M. microtis* is more effective than that of *P. discolor*. Hence, *M. microtis* has a least two adaptations to hunting in cluttered environments: using higher frequencies [29] and a noseleaf that is relatively long compared to the wavelengths in its call.

Current models of bat echolocation typically break down when fed signals in which many overlapping echoes are present simultaneously, e.g. SCAT model [37]. Moreover, at least some FM bats have been documented to change their hunting strategy in highly cluttered environments, switching from detecting of food by echolocation to passive hearing of, for instance, rustling noises produced by the prey itself [28], [38]. Hence, we conclude that extracting echoes of interest from signals containing many overlapping echoes is a difficult segmentation problem in echolocation that could be partially addressed by having a spatial selection mechanism such as a noseleaf. Finally, our data indicate that, by moving the noseleaf, the bat has some control over the region in which it focuses its energy. Hence, the bat can shift this spatial focal area to different regions of interest depending on task demands.

### Ecological Relevance of the Inter-specific Differences

The lancet of *M. microtis* is larger with respect to the wavelengths it uses. As we show, this results in an improved focusing of the radiated energy and potentially better control over the emission beam due to lancet bending and additional localization cues. We propose that the differences in functionality of the noseleaf between the two species reflect the ecological differences between the two species.

*M. microtis* is a gleaning bat that hunts at close range [30] and mostly preys on rather large insects that frequently sit motionless on or at leaves or branches in the forest [31]. Indeed, its broadband call is very suited for this task [29]. Behavioral studies have shown that *M. microtis* hunts by flying up and down the vegetation thereby checking every leaf for potential prey [30]. Consequently, this bat is mostly interested in inspecting a small portion of the world with each call as such a strategy improves detection and classification of motionless prey sitting on leaves by reducing background clutter. Additionally, bending the noseleaf allows for finer control of the energy distribution over space. Finally, focusing the emitted energy also reduces the probability of eavesdropping by potential prey that is outside the focal area. Therefore, focusing the energy in a small focal area makes sense when considered against the sensorial and ecological background of the animal where it faces the challenge to discriminate between echoes from prey and echoes from the surrounding vegetation.

In contrast, *P. discolor* feeds on a wide range of foods [32] that can often be found through olfaction or vision [27], [32]. Our finding that this species' noseleaf focuses the energy to a lesser extent and introduces no spectral localization cues reflects a lesser degree of sensorial specialization where echolocation is mostly used for orientation in space and other cues, in particular scent, for detection of food. This species uses a noseleaf that is smaller compared to the used wavelengths and its body size. Thus it seems that the feeding niche of this species does not require the evolution of a larger noseleaf and increased focusing. Apparently, a larger noseleaf does not yield a net advantage for *P. discolor*.

It remains an open question how the current results can be extended to other species. The bats included in this study are extreme examples with respect to their feeding ecologies. *M. microtis* is very specialized while *P. discolor* is a generalist. Although it is somewhat dangerous to draw firm conclusions on the basis of only two cases, we expect most other species to fall somewhere in between these two extrema with respect to the ratio between the noseleaf length and the used wavelengths. The species-rich, endemic family of New World leaf-nosed bats (Phyllostomidae) offers exciting opportunities for such studies with more than 175 species that vary widely in their ecologies and morphologies. The dietary spectrum includes blood, small vertebrates and insects as well as fruits, pollen, nectar and in part also leaves. Noseleaves range from the very long (several cm) lancet in the insectivorous *Lonchorhina aurita*) to tiny noseleaves in nectar-drinking bats or a total reduction as in the blood-drinking vampire bats (i.e., *Desmodus rotundus*) or the fruit-eating bat *Centurio senex*.

### Conclusion

We conclude that the noseleaves in both FM bats studied here serve mostly to enhance the signal to noise ratio in an area of interest to the bats. A direct effect of this is that the bats ensonify objects outside of this region to a lesser extent. Therefore, we propose that the noseleaf increases the strength of the signals returning from objects of interest to enable the ears to imprint reliable localization cues upon the returning echoes. The effect of the lancet on the spatial distribution of the energy is larger in *M. microtis* than in *P. discolor*.

Moreover, the lancet of *M. microtis* potentially plays a functional role in shaping the spectra of the total sonar system by enhancing potential cues for localizing targets. In contrast, the lancet of *P. discolor* introduces almost no additional cues in the spatial sensitivity pattern of the complete sonar system.

The differences we found between the two species can be interpreted mostly in terms of their different feeding ecologies. Therefore, we propose that the main functionality of the lancet in each species of bat reflects a specific adaptation to its feeding niche.

## Materials and Methods

Among the computational methods, Boundary Element Methods (BEM) are well suited for simulating the HRTFs of complete heads [18], [19], [39], [40]. In our lab, BEM has previously been used and validated to simulate the HRTF of bats using *P. discolor* as a model [19]. Additional validation of BEM for calculating HRTFs in the ultrasonic domain stems from the comparison between simulations and measurements in gerbils [40].

Simulating the HRTF or emission beam pattern of a bat using BEM requires building a 3D mesh model of the specimen's head surface. The method used in this study to obtain such models has been reported by [19] in detail. It consists of three main steps: (1) data acquisition using micro-CT, (2) segmenting an initial model and (3) simplifying the model.

The heads of one specimen of *M. microtis* and one specimen of *P. discolor* were scanned using a Skyscan 1076 micro-CT machine (http://www.skyscan.be/products/1076.htm) at a resolution of 35 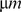. The CT shadow images were reconstructed using the software provided by the manufacturer yielding a set of grayscale images. These data were downsampled to a resolution of 70 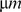.

Using standard biomedical imaging software (Amira, http://www.amiravis.com/), the 3D voxel data were segmented by separating the tissue from the background. This was done automatically as much as possible. However, some manual corrections were necessary. From the segmented data an initial mesh model was rendered. The acoustic simulation environment allows simulating models only up to about 32,000 triangles. Therefore, we had to simplify the initial models. Several rounds of smoothing and remeshing the model decreased the number of triangles to less than 32,000. These steps mainly reduced the surface area of the model (reduction of about 15 percent) while keeping the volume constant (reduction was less than 1 percent), indicating that simplifying the mesh mainly reduced the noise. The resulting mesh models consisted of triangles with a largest edge length of 0.5 mm. At the highest frequency employed in this study (150 kHz, see below) this is less than 0.25th of the wavelength.

The simulations of the HRTF were run on the CalcUA (http://www.calcua.ua.ac.be), a computer cluster consisting of 256 nodes running BEM3D acoustic simulation software [18], [39], [41]. Multiple (4) virtual receivers were positioned in both the left and the right ear at the entrance of the ear canal [19]. The sound field was averaged across the receivers for each ear separately. Additionally, as reported in [22], one receiver was placed in each nostril of the models to simulate the emission pattern making use of the reciprocity principle [42]. Tests showed that the precise position of the receiver in the nostril opening does not affect the results significantly (see also [19] and references therein). The far field of the sound field for the emission was calculated by summing the complex pressure fields for both nostrils.

**Figure 12:**
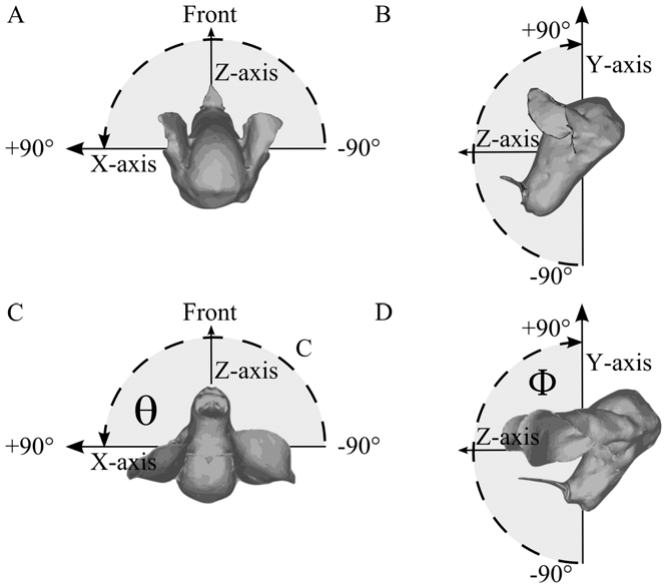
The coordinate system used in the simulations. Panel A and B: *P. discolor*, Panel C and D: *M. microtis*.

Sound sources were placed in the frontal hemisphere from −90 degrees to 90 degrees separated by 2.5 degrees in both azimuth and elevation at a distance of 1 m from the center of the models. Figure 12 illustrates the coordinate system used throughout this paper. Notice that both bat heads were positioned such that the plane in which the openings of the nostrils are located is perpendicular to the horizontal plane to ensure consistency with [3], [10], [11], [14], [19].

We assume that all the emitted sound energy stays within the frontal hemisphere i.e., negligible amounts of energy are radiated backward, requiring the normalization of the emission beam patterns of the bats per frequency 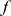


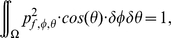
(1)with 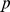 denoting the spatially dependent magnitude of the pressure for frequency 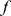 and 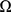 the frontal hemisphere. The spatial sensitivity of the complete sonar system is calculated by pointwise multiplication of the values at corresponding directions for the HRTF and the emission beam pattern at frequency 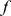 [5].

While we simulated both the HRTF of the left and the right ear for each bat, we only report data on one ear per bat as the right ear of the *P. discolor* as well as the left ear of *M. microtis* seemed slightly deformed due to the preservation of the specimen. We mirrored the data of the right ear of *M. microtis* to make the data congruent with that of *P. discolor*.

### Simulating the effect of clutter

To test the influence the lancets have on the reduction of clutter, we simulated the effect of 1 to 4 clutter echoes overlapping with a target echo using a Monte Carlo technique.

The attenuation of the clutter echoes by the sonar system is drawn randomly from the distribution of spatial energy as simulated with and without lancet outside a given center region. We varied the opening angle of the conical region we consider as the center region from 30 to 80. Furthermore, the strength of the clutter echoes was varied from −20 dB to 20 dB relative to the target echo. This models the possibility that the objects causing clutter echoes can be either better or worse reflectors than the target object. For each combination of number of clutter echoes, size of the central region and clutter echo strength we generated 1000 time signals composed of a target echo and a number of clutter echoes each with a random offset in time drawn uniformly from 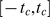 with 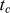 the duration of the call. For this ensemble of 1000 signals, we calculated the standard deviation of the resulting spectrum averaged across frequencies and number of clutter echoes. This is plotted in Figure 8.
